# Clinicians’ experiences of delivering online Community Reinforcement and Family Training (CRAFT) for families affected by substance use in rural Australia

**DOI:** 10.1016/j.dadr.2026.100416

**Published:** 2026-02-12

**Authors:** Subash Thapa, Heidi Gray, Nicole Snowdon, Brian Serna, Brianna Jacobson, Julaine Allan

**Affiliations:** a Rural Health Research Institute, Charles Sturt University, Orange, NSW 2800, Australia; b Serna Solutions, Santa Fe, NM, USA

**Keywords:** Community reinforcement and family training, Implementation science, Positive communication, Rural mental health, Substance-related disorders

## Abstract

**Background:**

Interventions that support family members or concerned significant others (CSOs) of people with alcohol or drug dependence can enhance recovery, strengthen family functioning, and reduce recurrence rates. Yet factors influencing successful implementation of CSO-focused online programs, especially in rural Australia remain poorly understood. This study explored the implementation experiences of clinicians delivering online Community Reinforcement and Family Training (CRAFT).

**Methods:**

This exploratory descriptive qualitative study involved semi-structured interviews with seven clinicians who delivered CRAFT. Data were analysed thematically using the RE-AIM (Reach, Effectiveness, Adoption, Implementation, Maintenance) framework.

**Results:**

CRAFT was perceived as well-structured, feasible, and compatible with routine clinical practice, enhancing clinicians’ confidence and enabling CSOs to develop skills in self-care, positive communication, problem-solving, and supporting healthy behaviours among loved ones. Successful implementation depended on clinician enthusiasm, flexible delivery modes (online as well as in-person), tailored content, and ongoing feedback, while challenges included CSO emotional overwhelm, competing responsibilities, limited digital literacy, and insufficient alcohol and drug services for loved ones. Socio-cultural factors, including reluctance to discuss alcohol or substance dependence, also limited engagement. Clinicians intended to retain key program components in practice but time constraints, telehealth limitations, and rural service gaps hindered sustained integration. Suggested improvements included cultural adaptation and structured opportunities for family–service collaboration.

**Conclusions:**

Online CRAFT is a promising, practice-ready intervention for supporting and empowering rural families affected by alcohol and drug use. Its integration into health services will depend on addressing workforce capacity, digital access, and rural service inequities, alongside policy-level commitment to family-focused programs.

**Clinical trial registration:**

ACTRN12623000796684 (registered 26 July 2023)

## Highlights

• First study of online family-focused alcohol support in rural Australia.
• Online Community Reinforcement and Family Training (CRAFT) was feasible and useful.
• Implementation depended on clinician enthusiasm, flexibility, and tailored delivery.

## 1 Introduction

Alcohol and substance use disorders represent a major public health challenge, affecting not only individuals with an alcohol and/or drug dependence but also their concerned significant others (CSOs), including family members, partners, ex-partners, or friends (Griswold and Karriker-Jaffe, 2025, Hellum et al., 2021). CSOs often experience increased emotional distress, financial strain, disrupted family functioning, and an increased risk of mental health conditions (McCann et al., 2019, Orford et al., 2010). CSOs of people with alcohol or drug dependence are particularly vulnerable, as exposure to harmful drinking or substance use within the household can lead to long-term psychological, physical, and social consequences (Giri et al., 2025, McCann et al., 2019). In Australia, approximately one in four adults report living with someone affected by problematic alcohol or drug use, which may negatively influence both individual mental health and overall family wellbeing (Teesson et al., 2010).

To support families affected by substance use, several CSO-focused interventions have been implemented internationally and in Australia, including psychoeducation, coping skills training, and structured therapeutic programs (Byrnes et al., 2010, Calabria et al., 2014, Geijer-Simpson et al., 2023, Kohlhoff et al., 2020, Pinto et al., 2024, Rowe et al., 2013, Ward et al., 2017). Evidence indicates that these interventions are effective in enhancing CSO coping strategies, reducing stress, and strengthening family functioning, but they have shown mixed results in reducing a loved one’s alcohol or substance use (Ariss and Fairbairn, 2020, Edgren et al., 2022, Geijer-Simpson et al., 2023, Ward et al., 2017). A scoping review highlighted that while implementing family-focused programs is essential, achieving high-quality delivery in community settings remains challenging (Pinto et al., 2024). These challenges often result from the interaction between program-related factors (e.g., program duration, provider competency) and contextual factors (e.g., community stigma, health system limitations) (Byrnes et al., 2010, Calabria et al., 2014, Kohlhoff et al., 2020, Pinto et al., 2024). Nevertheless, a multi-country trial demonstrated that implementation is feasible when supported by ongoing training, supervision, and provider enthusiasm, with preliminary evidence indicating effectiveness (Rowe et al., 2013).

Among family-focused programs, Community Reinforcement and Family Training (CRAFT) is an evidence-based approach that helps families reduce a loved one’s substance use, facilitate treatment engagement, and enhance family wellbeing through structured, personalised training and support. CRAFT has been tested and shown to be effective internationally (Archer et al., 2020, Ariss and Fairbairn, 2020, Calabria et al., 2020, Dopp et al., 2022, Hellum et al., 2023). Derived from Cognitive Behavioural Therapy (CBT) and Motivational Interviewing (MI), CRAFT is a structured six-session intervention that integrates theoretical learning with practical exercises (e.g., problem-solving, communication skills, and reflective activities), can be delivered either in person or online, using tailored approaches supported by ongoing supervision and feedback (Allan et al., 2024). Between September 2023 and November 2024, CRAFT was implemented online in rural Australia, providing a CSO-focused therapy model for alcohol and substance use in rural Australia.

Few CSO-focused support programs, such as CRAFT, are offered in rural Australia, and these programs often lack systematic evaluation of factors that influence their delivery, adoption, fidelity, and integration in routine practice (Calabria et al., 2014, Kohlhoff et al., 2020). Understanding the implementation factors surrounding such programs in rural settings is critical to ensuring that they not only achieve their intended outcomes but are accessible to CSOs who need them most. Therefore, this study aimed to explore clinician experiences of learning and implementing online practitioner-led CRAFT using the RE-AIM (Reach, Effectiveness, Adoption, Implementation, and Maintenance) framework, to identify facilitators, barriers, and opportunities to optimise program delivery, inform practice improvements, and support integration into mainstream rural health services.

## 2 Methods

### 2.1 Study design

This study opted for an exploratory, descriptive qualitative (EDQ) study design to explore implementation of CRAFT. EDQ provides straightforward, low-inference descriptions of clinicians’ experiences and is recommended when the goal is to characterise poorly understood phenomena (barriers and facilitators) from implementers’ perspectives (Hunter et al., 2019). Additionally, framing data collection and analysis with the RE-AIM framework further strengthens the approach by supplying a pragmatic, implementation-focused structure that is widely used to tailor evaluations to clinical and community settings and to link rich qualitative findings to actionable implementation outcomes (Glasgow et al., 2019). Ethical approval for this study was obtained from the Charles Sturt University Human Research Ethics Committee (Reg no. H23769).

### 2.2 Study settings

This study was a sub-study of a randomised controlled trial (RCT) evaluating the effectiveness and impact of online CRAFT and included interviews with the clinicians delivering the program to the participating CSOs (Gray et al., 2026). CSOs involved in CRAFT were aged 18 years or older and had a relative or loved one with a substance use disorder, with or without a co-occurring mental health condition and from rural areas (Modified Monash Model 2–7) were included in the program. The Modified Monash Model is used by the Australian Department of Health and Aged Care to define whether a location is classified as metropolitan, rural, remote, or very remote (Versace et al., 2021). Categories range from MM1 (major city) to MM7 (very remote) and for this trial, rural Australians were considered anyone who lived in MM2 to MM7.

### 2.3 CRAFT intervention

The intervention was delivered across six weekly 60-minute sessions using the *Family Empowerment Program Brief Facilitator Guide* (Allan and Snowdon, 2024). This guide was adapted from the original CRAFT materials (Smith and Meyers, 2022) and developed by an accredited CRAFT therapist, supervisor and trainer (JA). Further details of the CRAFT intervention, including an overview of session content, are reported elsewhere (Gray et al., 2026).

### 2.4 Clinician training and accreditation process

Flyers outlining the program and research project were sent to practice principals and team leaders. Interested clinicians were asked to contact the project leader (JA) to discuss the opportunity and participation requirements. A total of 18 people enrolled in the training workshop held in August 2023. Training consisted of 2 days of experiential sessions involving role plays and practice of the procedures delivered in-person by an accredited CRAFT trainer (BS). The participant training manual described all sessions and procedures that would be delivered in the CRAFT program. The manual was adapted from one written for an earlier study (Rose et al., 2014). Clinicians were required to attend both days of training and participate in accreditation and skills assessment to deliver CRAFT.

Accreditation consisted of providing audio recordings of individual sessions to an accredited CRAFT trainer and supervisor (BJ) for assessment, which followed a structured assessment guide provided by the accrediting organisation. If assessed as unsatisfactory, the clinician was required to resubmit the session until the required session elements were delivered with fidelity to the CRAFT program. When assessed as satisfactory, the clinician moved on to record and demonstrate the skills required for the next session of the program. Altogether, 10 clinicians agreed to deliver CRAFT and participate in certification, with six of these ten completing the accreditation process.

### 2.5 Participants, sampling and recruitment

Participants consisted of clinicians who had received formal training by accredited CRAFT trainers (BS, BJ) and were referred at least one study participant between October 2023 and November 2024. The study focused exclusively on clinicians who delivered the program, as they played a central role in the implementation and adaptation of CRAFT in real-world clinical settings. All clinicians were recruited from private psychology practices and drug and alcohol treatment services in eastern Australia. Participants were not required to have completed the accreditation process to be involved in the interviews.

For the recruitment of the clinicians, HG contacted all clinicians who had attended the training days and had at least one client referred to them. Seven clinicians agreed to interview. Of these seven, six had completed the training and accreditation process, while one had completed the training, had been referred participants but did not complete the accreditation.

### 2.6 Data collection

Online, semi-structured interviews were conducted between June 2024 and February 2025. The interview guide was developed in English by the research team, informed by the RE-AIM framework to capture key dimensions of **Reach** – who benefited and the extent to which they participated; **Effectiveness** – what was the key benefit intended; **Adoption** – what was actually achieved and who took up the intervention; **Implementation** – Extent to which the intervention was implemented as intended or was adapted; **Maintenance** – how strategies for sustainability were taken up and the intervention continued to be available (Holtrop et al., 2018).

To ensure conceptual clarity and relevance, the guide was reviewed by two qualitative researchers (H.G. and J.A.). Minor revisions were made to refine question wording and improve flow. The final guide included questions on participants’ experiences delivering CRAFT, perceived barriers to implementation, strategies for engagement and retention, adequacy of training, program feasibility, and recommendations for improvement.

All interviews were conducted by a trained qualitative researcher with experience in implementation studies (HG). To accommodate clinicians across diverse service locations, interviews were held via secure online video calls at times convenient to them. Written informed consent was obtained prior to the interviews, and clinicians were assured of confidentiality and the voluntary nature of participation. Interviews lasted between 25 and 55 mins. During the sessions, the researcher took field notes to capture non-verbal cues and key reflections. All interviews were audio-recorded with clinician permission.

A total of seven clinicians agreed to participate in the study. Six participants worked in private practice and one participant worked in a community health service. Out of the seven participants, one clinician stopped providing CRAFT during the trial and therefore did not become accredited (see Table 1).

**Table 1:** Characteristics of participants.

**Name (pseudonym)**	**Age (years)**	**Gender**	**Professional background**	**Work experience (years)**	**Gained accreditation**
Sharon	> 50	F	Master of Psychology	1–5	Yes
Deb	> 50	F	Bachelor of Psychology	21–25	Yes
Janet	> 50	F	Graduate Diploma in Psychology	1–5	Yes
Rob	30–39	M	Bachelor of psychology	6–10	No
Maggie	40–49	F	Bachelor of Psychology	6–10	Yes
Linda	> 50	F	Master of Psychology	16–20	Yes
Pat	40–49	F	Bachelor of Psychology	11–15	Yes

### 2.7 Data analysis

All interviews were audio-recorded, transcribed verbatim, and checked for accuracy. Thematic analysis was conducted using both deductive and inductive approaches, guided by the RE-AIM framework. Initially, a deductive coding structure based on the five RE-AIM dimensions was applied to organize the data. This was followed by inductive coding to capture new themes (e.g., contextual factors) that emerged beyond the predefined framework. Codes and subthemes were iteratively refined through multiple discussions among the research team to ensure consistency and credibility. The clinicians’ own words were used to illustrate key findings and maintain contextual richness. NVivo 15 software was used to support the analysis.

### 2.8 Controlling for the quality of the study

H.G., a female researcher trained in qualitative methods, maintained regular contact with clinicians and conducted all participant interviews. She took deliberate steps to remain neutral and reflexive, including documenting reflections after each interview and considering how her positionality might influence participant interactions. S.T., a mental health researcher with expertise in qualitative research methods, conducted data analysis. To ensure credibility and consistency, the study team held regular discussions about preliminary findings, reflecting on how the professional backgrounds and assumptions of H.G. and S.T. might shape interpretation. These reflexive dialogues helped identify potential biases and alternative explanations. B.S. and B.J., researchers from a different healthcare and social context, reviewed emerging themes and offered external interpretations. Their distance from the study context enabled them to challenge possible over- or under-interpretations and supported the conceptual framing of the findings. Together, these processes enhanced the study’s trustworthiness and methodological rigour.

## 3 Results

Our analysis identified themes across all five RE-AIM domains (reach, effectiveness, adoption, implementation, and maintenance), encompassing both program-related components and broader contextual factors influencing engagement, implementation outcomes and sustainability (see Table 2).

**Table 2:** Coding table.

**Categories**	**Broader codes**	**Codes**
Reach	Client characteristics, readiness & Engagement	Low program awareness among community and referrers; Stigma surrounding substance use; Reluctance to discuss alcohol-related issues; Denial or low perceived need for help; Perceived lack of relevance (invited by others); Low perceived value due to free service; Caregiving challenges and competing responsibilities; Lack of emotional and cognitive readiness to engage; Client motivation fluctuating by loved one’s treatment stage; Closeness or contact with family member using substances influencing engagement; Cultural stigma among First Nations clients; Low eHealth literacy and technology confidence; Transport and distance issues in rural and remote settings.
	Complexity of Client Needs	Diverse CSO profiles (partners, parents, adult children); Co-occurring mental health conditions (trauma, ADHD, anxiety, depression); Long history of familial substance use (chronic patterns over decades); Varied stages of change across families; Unfulfilled expectations of program outcomes; CSOs’ own substance use; Need for trauma-informed and motivational interviewing approaches; Clients’ caregiving stress and emotional exhaustion; Intergenerational dynamics complicating engagement.
Effectiveness	Program Feasibility	Program perceived as structured, feasible, and compatible with counselling practice; Enhanced clinician confidence; Instilled hope among CSOs; Reinforced motivation through observed progress; Rewarding for counsellors to see “aha moments”; Easy to integrate within existing caseloads; Variability in attendance and completion due to family stressors; Emotional readiness, trauma, and ADHD influencing engagement and outcomes; Feasible but limited by time and CSO mental health challenges.
	Program Delivery Flexibility	Flexible in delivery (face-to-face and telehealth); Manualised content manageable when adapted; Early sessions overloaded with content; One-hour sessions often insufficient; Weekly scheduling conflicts and rescheduling issues; Clients preferred slower pacing or extended time; Challenges sustaining continuity across weeks; Need for simplified or redistributive session structure; Telehealth increased access but required adaptation for low digital literacy.
	Perceived Effective Components	Communication and problem-solving modules highly valued; CBT-aligned structure improved understanding; Encouraged self-reflection and boundary-setting; Provided psychoeducation and coping tools; Tailored feedback and examples improved engagement; Telehealth acceptable when supported; Supervision, peer feedback, and clinician enthusiasm enhanced effectiveness; Visual aids and examples improved comprehension; Goal setting and positive reinforcement strategies useful but dependent on loved one’s engagement.
Adoption	Clinician Capacity & Workload	Clinician workload pressures, competing priorities and time scarcity; Performance anxiety regarding manual fidelity; Fear of “doing it wrong”; Prior alcohol/substance use work experience increased confidence; New counsellors faced initial uncertainty; Funding and staffing constraints affected recruitment; Clinician inactivity in regional services; Emotional fatigue; Burnout concerns; Workload limited session preparation; Challenges engaging clients amid competing caseloads.
	Adaptation Strategies	Adapted program for cultural fit and literacy; Tailored examples for rural and First Nations clients; Used visual aids (whiteboard, screen sharing); Adjusted pacing and order of modules; Extended or shortened sessions based on client needs; Added extra sessions when needed; Maintained fidelity while increasing flexibility; Emphasized practical examples; Integrated motivational interviewing; Supported clients through supplementary notes or summaries.
	Training & Supervision Gaps	Training well-balanced between theory and practice; Role-plays highly valuable; Limited guidance in initial sessions; Anxiety about adhering to manual; Ongoing supervision reinforced confidence; Peer support encouraged reflection; Prior CBT experience was beneficial; Desire for more examples and local case discussions; Need for structured feedback loops post-training.
Implementation	Program Structure & Delivery Challenges	Structured six-session format aligned with CBT; Session length constraints (1-hour limit); Overloaded early sessions (especially session 1); Manualised structure occasionally perceived as rigid; Variability in client readiness hindered progress; Emotional overwhelm among CSOs; Challenges maintaining engagement across telehealth sessions; Rural and remote clients’ digital barriers; Limited local resource knowledge for referrals; Challenges synchronizing with CSO availability; Scheduling challenges for working caregivers
	Contextual and System-level Barriers	Limited local treatment services for referrals; Gaps in regional service networks; Digital access and literacy challenges; Limited awareness among service providers; Fragmented service coordination; Insufficient local knowledge among telehealth clinicians; CSOs’ competing family and work demands; Socio-cultural barriers to help-seeking; Technology fatigue during telehealth; Lack of peer support for counsellors.
	Implementation enablers	Clinician enthusiasm and reflective practice; Flexibility in adapting delivery modes; Use of visual tools and shared screens; Feedback loops between sessions; Client progress monitoring; Use of empathic communication and active listening; Tailoring pace to client readiness; Adjusting examples to cultural context; Practical tools reinforcing learning; Collaborative goal setting; Blending online and face-to-face sessions effectively.
Maintenance	Sustainability Enablers	Ongoing use of program components in counselling; Increased confidence with repeated delivery; Integration into routine counselling workflows; Structured modules seen as adaptable templates; Counsellors continuing to use materials flexibly; Positive clinician attitudes toward future implementation; Desire to integrate with broader family programs; Relevance to other CSOs and non-AOD clients; Feasibility for long-term embedding with modest resource support.
	Sustainability Barriers	Limited resources and time for extended implementation; Rural service constraints and staff turnover; Lack of long-term funding mechanisms; Ongoing recruitment challenges; High client demands limiting completion; Systemic gaps in regional service infrastructure; Absence of organizational policy support for sustained delivery; Unclear referral pathways; Overreliance on individual clinician enthusiasm; Lack of ongoing supervision post-trial.
	Psychosocial and Cultural Challenges	CSOs’ emotional overwhelm and distress; Guilt and shame discussing family substance use; Denial or avoidance behaviours; Women’s dual caregiving and domestic responsibilities; Barriers among Aboriginal and First Nations CSOs; Stigma and confidentiality concerns in small towns; Limited culturally tailored materials; Socio-cultural norms affecting disclosure; Need for culturally safe adaptation and trauma-informed facilitation.

*ADHD – Attention deficit hyperactivity disorder; AOD – Alcohol and other drugs; CBT – Cognitive behavioural therapy; CSOs – Concerned significant others; eHealth – Electronic health

### 3.1 Reach: who the program engaged and how

Several clinicians mentioned that client recruitment was unpredictable and that they were not sure when referrals would come in. This was attributed to limited awareness of the program, stigma surrounding substance use, and challenges in identifying and engaging families affected by alcohol and drug dependence, particularly in rural and remote areas. One clinician stated:

*“If they were caring for a loved one who’d been drinking for 30 years, change is pretty much unlikely, right. Now, that didn’t mean that there wasn’t anywhere to go with it. Because there was a different focus I guess on the carer’s wellbeing.” [Sharon]*

Among the clinicians, those with previous experience in alcohol and substance use work found it easier to follow the program’s structure than those without such experience. Most clinicians agreed that the timing of program delivery and client readiness were critical for engagement. CSOs ready to change were enthusiastic and followed the sessions effectively, whereas those with limited contact with their loved one had fewer opportunities to apply the program techniques.

*“For others, it was just never going to be the right time when we were doing it. And I kept reinforcing that, that it’ll just happen when it happens, and you’ll be armed with the information to do something.” [Deb]*

Some CRAFT participants were described as denying the psychological challenges of substance use or were not in frequent contact with the person with the substance use disorder so were perceived as less interested in the session content. One clinician explained that some CSOs didn’t feel the need to apply the program techniques because they were invited to take part by other family or friends rather than seeking support out themselves. These participants were also perceived to place less value on CRAFT since the sessions were offered for free.

### 3.2 Effectiveness: perceived benefit and outcomes

The clinicians described the CRAFT program as well-structured, feasible, and compatible with existing counselling practices. It was perceived to enhance their confidence in managing CSOs with complex family dynamics. They noted that the program provided opportunities for CSOs to enhance coping and communication skills, improve decision making, and increase self-awareness. Observing their CSOs making progress reinforced the clinician’s motivation and perceived value of delivering the sessions.

*“But what I found really cool was when they had ‘ah-ha’ moments …And how helping their loved one, they were helping themselves by setting boundaries in place and stopping things from triggering them. Yeah, that was good.” [Janet]*

The clinicians stated that although CSOs generally found the program engaging, challenges with attendance and completion persisted due to scheduling conflicts and personal stressors. For example, Sharon said “if you’ve got someone that’s a little bit apprehensive or really busy or has their own mental health conditions, then spreading it out might be better for them. They need a more time to kind of process what was said during the session, a little bit more time to complete the homework”.

Several clinicians reported that their CSOs were initially uncertain about the program’s benefits, particularly those coping with long-term family substance use (over 30 years) who had not previously experienced any effective intervention. However, their experience with CRAFT changed this

*“What I loved about it was the situations were so dire for their loved ones, so long standing, there was not much hope. But what is wonderful, was to know that their relationship had changed with their loved one. That was really beautiful. [Deb]*

### 3.3 Adoption: uptake by clinicians and service settings

The clinicians emphasized the importance of the initial training, noting that it was well-structured and balanced between theory and practical exercises. They highlighted that participating in role plays was highly valuable for their real-life practice. The skills were perceived as transferable, benefiting work with both families affected by alcohol and drug use and other CSOs. However, one clinician mentioned that it took a while for her to become accustomed to session contents and felt that “*the lack of detailed guidance for each session made it initially challenging.*“ [Maggie].

Some clinicians expressed anxiety about adhering strictly to the manualised components of the program and needing to demonstrate them in recordings that were sent for review by assessors. One clinician discontinued the program because of it:

*But then I think the pressure of the checklist and all that, I was like ‘What if I do it wrong?’ And it probably just caused a bit more anxiety than enjoyment.” [Rob]*

All the clinicians agreed that confidence and ease improved with practice and experience over time. Confidence to complete the sessions effectively seemed to depend also on clinicians’ prior experience, and time availability. The clinicians noted that CSOs’ engagement was facilitated by empathetic communication, and perceived benefits of supporting their loved ones or improving their own wellbeing. They also reflected on the variability in client readiness and engagement, highlighting the challenges of delivering sessions for some CSOs:

*“For some (CSOs) I was like ‘Oh, my goodness, I never thought you would [take the planned action with their loved one],’ but they did. And then some I could have sessions with them every week and I don’t know if they would have got it because they just weren’t in the place to receive it…. that’s what I found the hardest.” [Maggie]*

Two clinicians noted that the adoption of the positive reinforcement process depended on the involvement of the person using substances, which was difficult when that individual was not engaged regularly with the family from the outset.

### 3.4 Implementation: Delivery processes and contextual challenges

Most clinicians found that the program’s structured modules (e.g., communication, problem-solving) closely aligned with CBT principles they were experienced in and were practical to deliver within six sessions - *“A lot of the strategies, such as functional analysis, happiness scale, are ones that we do work [with] quite regularly [Rob].* This structure helped their CSOs engage and apply the strategies in their own contexts. The clinicians stated that, while some CSOs preferred less manualised content, most benefited from the structured modules when tailored to their needs.

The clinicians identified several key factors contributing to successful implementation, including flexibility in delivery modes (telehealth as well as face-to-face), the ability to adapt content based on client needs, provision of feedback and supplementary notes, clinician enthusiasm during sessions, and ongoing reflection on client progress.

*“I liked that it was very manualised but it was also flexible. You could do a lot of around it, there was time to do things around it. So, connecting with people and giving them a little bit of hope and teaching them that self-care was actually okay as well.” [Linda]*

The clinicians also mentioned several barriers to effective delivery, including CSOs becoming emotionally overwhelmed during sessions, reluctance to discuss familial substance use, and competing family or domestic responsibilities for the CSOs. Telehealth delivery brought both advantages and challenges. While it increased access, clinicians reported challenges engaging CSOs with limited experience in video calls. Some CSOs required assistance in accessing online sessions and completing between-session tasks, while some needed additional support to manage their mental health conditions.

*“Anyone that doesn’t have capacity for telehealth, it was definitely tricky. Because I’d get the whiteboard out most sessions, or you’d pull up the resource and be doing it together, so you’re sharing the screen. And I think that often the few that disengage because of the overload, they hadn’t managed how to work out telehealth.” [Pat]*

In addition, system-level barriers such as limited local treatment services to refer loved ones to further constrained online program delivery. For example, clinicians working on telehealth distant from the CSOs in small towns did not have local knowledge to share with their clients.

*“It could be a little bit overwhelming to discuss treatment and what services are available [when you’re not local]. And them not knowing and me saying about having to find what’s available in your area. [Deb]*

### 3.5 Maintenance: sustainability and future integration

All the clinicians expressed strong intentions to integrate core components of the program into their ongoing practice. Although there were anxieties in delivering the program initially, repeated delivery improved familiarity and adaptability, increasing their confidence to use the materials flexibly. One clinician stated,

*“I love the program. It was really helpful to always pull back on something that was written and in front of me. And it was interesting when the CSOs themselves had problems with alcohol. So, it felt like I was running on two different levels.” [Janet]*

However, ongoing integration was hindered by practical barriers such as how they would attract referrals for family members seeking support around substance use: *“I got sent the people because of the research project, I haven’t had these types of referrals before.” [Maggie].* Further, the clinicians had suggestions for program changes to improve delivery including extending session duration or redistributing content to allow more time for engagement.

*“if there was any dropout, it could be because there’s too much in the first session. And I think that they (CSOs) need more time to be heard.” [Deb].*

Several indicated that 6 weeks was not enough time for the whole program. They also suggested cultural tailoring for First Nations people and women experiencing socio-cultural challenges such as domestic violence. For example, one clinician stated: *“Asking what her cultural experiences were and her not being able to join because of the violence and things that have happened, it was a real eye-opener for me” [Sharon].*

## 4 Discussion

To our knowledge, this is the first study reporting clinicians’ experiences of implementing an online family-focused support program for individuals with substance dependence in rural Australia. This study found that CRAFT was well received in terms of acceptability and practicality, with trained clinicians actively engaging in program delivery and wiling to apply strategies in their own practice. The program’s structured yet flexible design, opportunities for CSOs to reflect on their circumstances, and tailored support were identified as key facilitators of engagement. CSOs who recognized a personal need for support and had adequate telehealth literacy were percieved to engage effectively with the program. Barriers to successful delivery included clinician performance anxiety, and CSO’s other personal and family demands. Recruitment and retention were further constrained by stigma and discomfort discussing underlying issues such as CSO substance use or family violence, particularly in rural contexts.

Unlike previous research primarily focused on program efficacy on client substance use behaviours (Calabria et al., 2014, Ward et al., 2017), this study offers a distinctive contribution by examining the real‑world implementation of a family‑focused intervention to empower family members within rural Australian service contexts. Implementation challenges also differ in these programs. For example: family focused interventions targeting persons with substance use disorders highlighted provider-factors such as clinical certification, and technical supervision challenges (Byrnes et al., 2010, Calabria et al., 2014, Kohlhoff et al., 2020). Whereas the current study identified barriers related to the target group, such as CSO readiness and experience with online communications, and socio-cultural sensitivities around domestic violence in families for example, that influence program adoption and sustained engagement.

Given that one in three individuals with a substance use disorder also has a co-occurring mental health disorder in Australia (Australian Institute of Health and Welfare, 2022), families often carry a disproportionate share of responsibilities in such circumstances, facing increased care responsibilities, stress, and family conflict (Lindt et al., 2020, McCarthy et al., 2022, Mihan et al., 2023). However, current mental health services are predominantly concentrated in major cities, meeting only around one-third of the total demand, and only 7 % of the 139,300 clients receiving treatment in 2021 were family members or friends of people with a substance use disorder (Health and Welfare, 2023). Given the shortage of rural services, online delivery of CRAFT provides a feasible and scalable model to reach families who otherwise lack access to care (Health and Welfare, 2023). Integrating CRAFT within rural health services could bridge a critical service gap by equipping families with the knowledge and skills to support recovery, reduce stigma, and improve wellbeing in underserved communities.

Our study highlights the ways behavioural intervention programs can apply cultural adaptation to rural settings and lifestyles, use telehealth infrastructure, and offer clinician training and fidelity to support the effective delivery of evidence-based interventions, aligning with existing evidence on the implementation of family-based interventions (Byrnes et al., 2010, Calabria et al., 2014, Kohlhoff et al., 2020). Embedding CRAFT within rural telehealth service structures should be supported by dedicated funding, investment in digital infrastructure and digital literacy programs, regular clinician training and ongoing supervision (Thapa et al., 2023). Support networks for clinicians working in geographically isolated areas are also essential to reduce burnout and ensure program fidelity.

This study has several limitations. First, the sample size was small, with only seven participants, which may limit both the depth of insights captured and the overall transferability of the findings. Second, data were collected exclusively from clinicians involved in a structured trial, which may not fully represent experiences in routine service delivery. Third, the exploratory descriptive qualitative design, while valuable for generating in-depth insights, may not capture the full diversity of perspectives among CSOs or contextual nuances across service settings. As a result, transferability to other populations or service contexts is limited. Last, only one clinician who discontinued the program was interviewed. Those who did not deliver CRAFT in the trial were mostly not represented, possibly biasing findings toward positive experiences.

Despite these limitations, the study provides important insights into how structured family-focused interventions can be effectively implemented and sustained in rural Australian contexts. Future research should include perspectives from CSOs, explore long-term outcomes, and assess how program integration within rural and regional health systems influences sustainability and service equity.

## 5 Conclusion

The online CRAFT program was perceived as well-structured, feasible, and compatible with routine clinical practice, supporting CSOs in developing skills in self-care, communication, decision making, and promoting healthy behaviours. Successful implementation was facilitated by flexible delivery modes, tailored content, clinician enthusiasm, and ongoing feedback, while barriers included emotional overwhelm during sessions, limited experience in online communications for healthcare, and rural service gaps. Policy and practice efforts should prioritize cultural adaptations of the program, enhance telehealth access, and provide sustained training and funding for clinicians. Targeted policy strategies are needed to optimize the implementation and accessibility of family-focused interventions in rural and underserved communities.

## CRediT authorship contribution statement

**Julaine Allan:** Writing – review & editing, Supervision, Methodology, Investigation, Formal analysis, Conceptualization. **Brianna Jacobson:** Writing – review & editing. **Brian Serna:** Writing – review & editing. **Nicole Snowdon:** Writing – review & editing, Validation, Methodology, Investigation. **Heidi Gray:** Writing – review & editing, Validation, Investigation. **Subash Thapa:** Writing – original draft, Software, Methodology, Investigation, Formal analysis.

## Ethics and consent

Ethical approval for this study was obtained from the Charles Sturt University Human Research Ethics Committee (Reg no. H23769). All participants provided their written informed consent for their participation in the study.

## Declaration of Generative AI and AI-assisted technologies in the writing process

*During the preparation of this work the author(s) did not use any AI or AI-assisted technologies. All the author(s) have reviewed and edited the content as needed and take(s) full responsibility for the content of the published article.*

## Funding

This work was supported by the Commonwealth of Australia, represented by the Department of Health and Aged care (Grant Activity 4-DGEJZ1O/4-CW7UT14).

## Declaration of Competing Interest

The authors declare that they have no known competing financial interests or personal relationships that could have appeared to influence the work reported in this paper.

## Data Availability

None (We do not share data due to the confidentiality of the participants and the requirements of our HREC approvals).
